# Idiopathic esophageal intramural hematoma associated with hemothorax leading to esophageal perforation

**DOI:** 10.1007/s12328-026-02321-4

**Published:** 2026-04-03

**Authors:** Atsushi Gakuhara, Ryugo Teranishi, Kurumi Tsuchihashi, Soichiro Minami, Wataru Fujii, Chikato Koga, Naotsugu Haraguchi, Jin-ichi Hida, Tomoko Wakasa, Yutaka Kimura

**Affiliations:** 1 Department of Gastroenterological Surgery, Kindai University Nara Hospital, 1248-1, Otoda-cho, Ikoma City, Nara, 630-0293 Japan; 2 Department of Pathology, Kindai University Nara Hospital, 1248-1, Otoda-cho, Ikoma City, Nara, 630-0293 Japan

**Keywords:** Idiopathic esophageal intramural hematoma, Hemothorax, Esophageal perforation

## Abstract

Idiopathic intramural hematoma of the esophagus is a rare condition associated with Mallory–Weiss syndrome or spontaneous esophageal rupture. It is usually manageable with conservative treatment but, in rare cases, may be complicated by hemothorax or progress to esophageal perforation. We report the case of a 56-year-old man with right-sided paralysis following a stroke who was taking aspirin. He presented with chest pain, a suspected mediastinal tumor, and right pleural effusion on CT. At our hospital, contrast-enhanced CT suggested a mediastinal hematoma with contrast leakage. He had no hematemesis or vomiting, and his vital signs were stable. On the fourth hospital day, he developed tachycardia and inflammatory changes. CT revealed pneumothorax, and oral contrast confirmed leakage from the esophagus into the right thoracic cavity. Emergency surgery revealed a large bloody pleural effusion, hematoma, and a 4-cm longitudinal tear of the esophageal muscular layer. Esophagectomy with cervical esophageal fistula and gastrostomy was performed. Pathological examination demonstrated a hematoma extending from the adventitia to the muscularis propria, with an acute, well-defined tear and no neoplastic lesion, consistent with idiopathic intramural hematoma. We report a rare case of idiopathic esophageal intramural hematoma with hemothorax leading to esophageal perforation, successfully treated by surgical intervention.

## Introduction

Idiopathic intramural hematoma of the esophagus (IEH) is a rare condition characterized by bleeding within the esophageal wall without mucosal rupture [1]. In contrast to Mallory–Weiss syndrome, which involves mucosal lacerations, and Boerhaave syndrome, which involves full-thickness perforation, IEH is defined by blood accumulation between the layers of the esophageal wall. IEH can be classified into two types: mechanical (caused by trauma, instrumentation, or foreign bodies) and idiopathic (often associated with increased intraesophageal pressure from vomiting or dietary factors) [2, 3]. Underlying coagulation disorders and the use of antiplatelet or anticoagulant drugs may also be present [4]. Typical symptoms include chest pain, dysphagia, and hematemesis [5]. The condition is usually reported to be manageable with conservative treatment; however, in rare cases, it may progress to perforation of the thoracic cavity with hemothorax [4, 6–9]. Cases resulting in further perforation are extremely uncommon.

## Case report

**Fig. 1:**
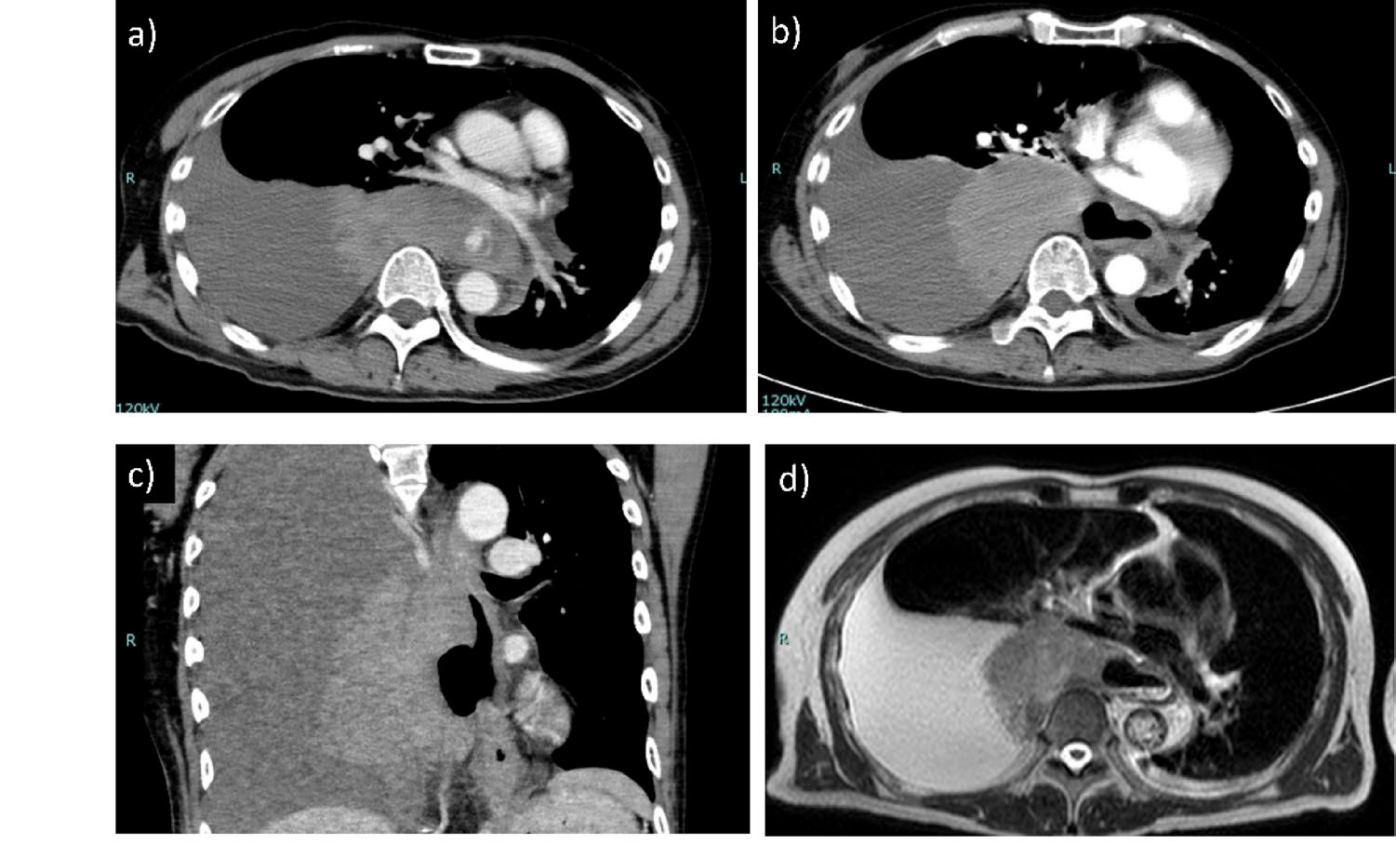
**a** Contrast-enhanced chest CT showing a mediastinal mass-like lesion along the mid-thoracic esophagus with right pleural effusion. **b**, **c** Contrast-enhanced chest CT demonstrating a diverticulum-like lesion in the right wall of the mid-thoracic esophagus. **d** T2-weighted MRI showing a homogeneous high-intensity lesion along the esophageal wall, consistent with an intramural hematoma

**Fig. 2:**
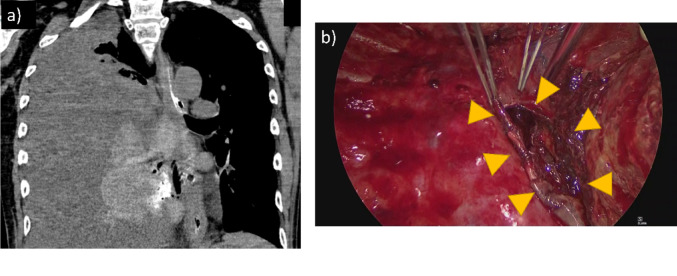
**a** Chest CT on day 4 after admission showing extravasation of contrast medium from the mid-thoracic esophagus into the right pleural cavity. **b** Intraoperative findings showing a 4-cm longitudinal tear of the esophageal muscular layer in the mid-thoracic esophagus (yellow arrows)

**Fig. 3:**
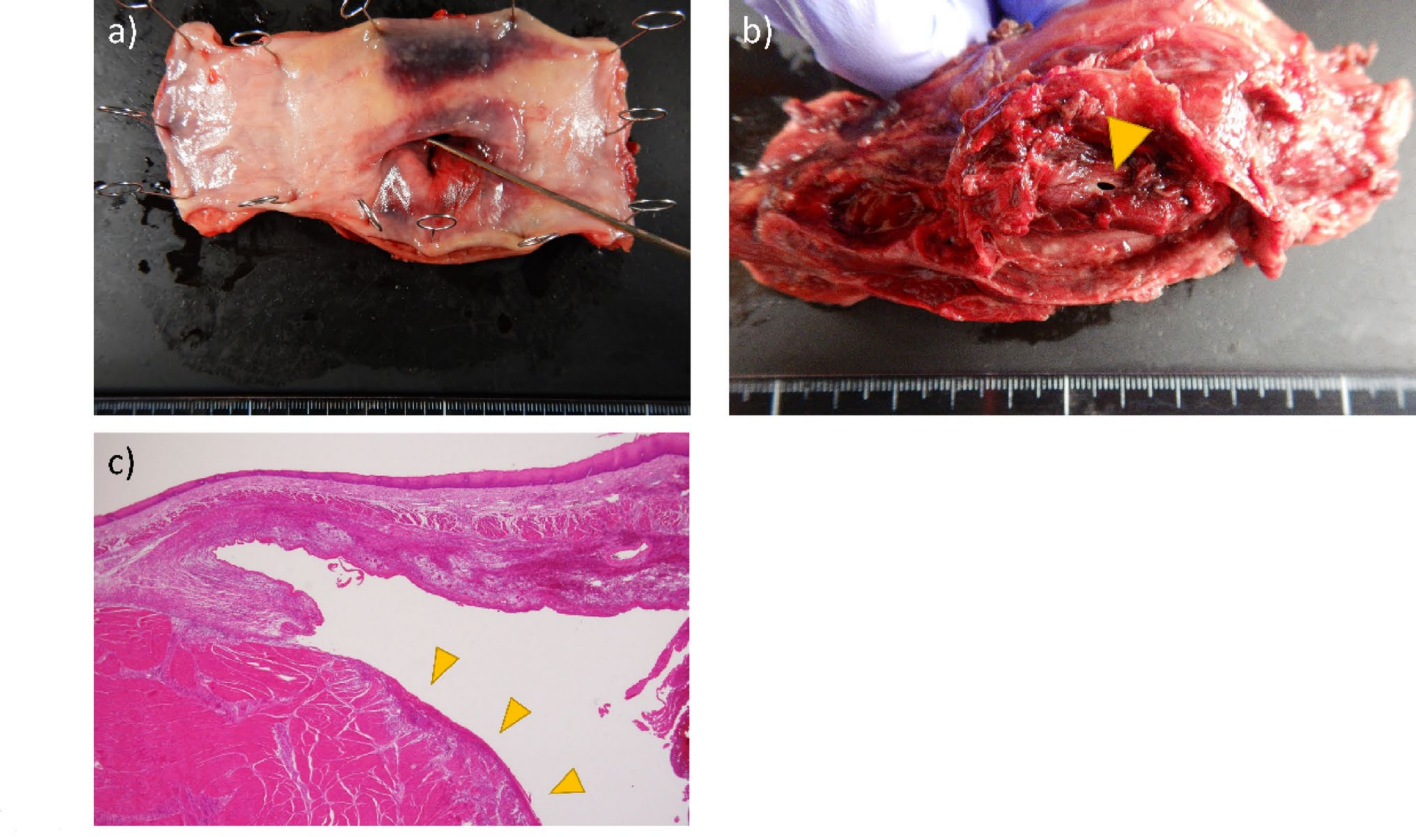
**a** Resected specimen (luminal side) showing a small pinhole-like mucosal defect confirmed by probe insertion. **b** Resected specimen (serosal side) showing disruption of the muscular layer with a pinhole-like perforation after removal of the hematoma (yellow arrow). **c** Histopathological findings of the resected specimen showing sharp disruption of the muscular layer (yellow arrows). No significant fibrosis or chronic inflammatory changes are observed, suggesting an acute rupture of the esophageal wall (hematoxylin and eosin stain, × 100)

The patient was a 56-year-old man with right-sided paralysis following a cerebral infarction and was taking aspirin. He initially presented to his previous physician with chest pain. CT revealed a suspected mediastinal tumor and right pleural effusion, and he was subsequently referred to our hospital. On admission, the patient was hemodynamically stable, with a blood pressure of 124/88 mmHg, heart rate of 105 beats/min, body temperature of 37.4 °C, and oxygen saturation of 97% under nasal oxygen at 1 L/min. He had no symptoms such as vomiting, hematemesis, or cough, and no history of trauma. Contrast-enhanced CT suggested either a mediastinal tumor or a mediastinal hematoma and showed contrast leakage within the mass (Fig. 1a). No mediastinal emphysema or pneumothorax was observed. His vital signs were stable, and bleeding within the mass was suspected; therefore, he was admitted for observation in the ICU. CT performed on day 2 after admission showed no further contrast leakage within the mediastinal mass, suggesting that the bleeding had ceased. Progressive anemia required a blood transfusion. A diverticulum-like finding was observed in the right wall of the mid-thoracic esophagus at the level of the mediastinal mass (Fig. 1b, c). MRI T2-weighted images were also suspicious for mediastinal hematoma and showed diverticulum-like changes in the right esophageal wall, similar to the CT findings (Fig. 1d). Thoracentesis revealed bloody pleural effusion, but drainage was not performed because CT suggested that bleeding had stopped. As the patient’s condition remained stable, conservative treatment was continued without invasive endoscopy. During the subsequent clinical course, the patient gradually deteriorated. On hospital day 4, the patient developed marked tachycardia (heart rate 139 beats/min) and fever (38.2 °C), with blood pressure of 108/67 mmHg. Laboratory examination revealed a progressive decrease in hemoglobin level from 10.8 to 8.6 g/dL, consistent with ongoing blood loss associated with hemothorax. Inflammatory markers were also elevated during this period. Chest X-ray showed a pneumothorax, and CT revealed mediastinal emphysema and pneumothorax. Oral contrast demonstrated leakage from the esophagus into the right thoracic cavity (Fig. 2a). A diagnosis of esophageal perforation was made, and the patient was referred for emergency surgery. Right thoracotomy revealed a large bloody pleural effusion. Disruption of the mediastinal pleura of the middle mediastinum and a hematoma were present. A 4-cm longitudinal tear of the muscular layer of the mid-esophageal wall was observed (Fig. 2b). The exact site of mucosal perforation could not be identified intraoperatively, but the same area was thought to be involved. Inflammation was severe, making primary suture closure difficult. An esophagectomy with cervical esophageal fistula and gastrostomy was therefore performed. Examination of the resected specimen showed a tear of the muscular layer in the middle esophagus. There was no mucosal damage in the same area, but a pinhole-like perforation was seen in the stretched mucosa (Fig. 3a, b). No tumorous lesions were identified. Histopathological examination revealed a hematoma within the esophageal wall with sharp disruption of the intrinsic muscular layer, without evidence of fibrosis or chronic inflammatory changes, indicating an acute rupture (Fig. 3c). The hematoma predominantly involved the muscular layer, whereas the mucosa was largely preserved, except for a small pinhole-like perforation. No neoplastic lesion was identified, and a diagnosis of idiopathic esophageal intramural hematoma was made. It was considered that the idiopathic intramural hematoma had ruptured into the thoracic cavity, causing hemothorax and subsequent esophageal perforation. No postoperative pyothorax or other surgical site infection occurred. After nutritional support and rehabilitation, the patient underwent hand-assisted laparoscopic gastric tube reconstruction approximately one month after surgery. He experienced no postoperative complications and was transferred for rehabilitation on postoperative day 16.

## Discussion

Idiopathic intramural esophageal hematoma (IEH) is classically described as a submucosal lesion that most commonly involves the distal esophagus [1]. However, previous reports have demonstrated that cases complicated by hemothorax often show different anatomical characteristics, including involvement of the mid-thoracic esophagus and extension into the muscular layer [4, 6–9]. Although a diverticulum-like structure was suggested on imaging, upper gastrointestinal endoscopy performed approximately one year before symptom onset showed no esophageal diverticulum or structural abnormality. Therefore, a pre-existing diverticulum was considered unlikely. In the present case, the intramural hematoma appeared to rupture toward the thoracic cavity, while the mucosa remained largely intact. As a result, the stretched mucosa may have protruded outward, producing a diverticulum-like appearance. Thus, the diverticulum-like finding was interpreted as a secondary morphological change caused by deformation of the esophageal wall following rupture of the intramural hematoma, rather than a primary etiological factor.

Idiopathic intramural hematoma of the esophagus can be classified into two types: mechanical, caused by trauma, endoscopic procedures, or accidental ingestion of a foreign body; and idiopathic, resulting from increased intraesophageal pressure due to vomiting or dietary factors [1]. Some patients have a predisposition to hemorrhage, such as those taking antiplatelet or anticoagulant drugs or with underlying medical conditions, including cirrhosis, hematological disorders, or dialysis dependence [4]. Typical symptoms include chest pain, dysphagia, and hematemesis [5]. In the present case, there was no history of mechanical injury or vomiting. The patient was taking aspirin, an antiplatelet drug that may have predisposed him to developing idiopathic intramural hematoma of the esophagus. Symptoms included chest pain but not hematemesis, consistent with the absence of disruption of the esophageal lumen. This lack of hematemesis was one reason the diagnosis was challenging.

The differential diagnosis of idiopathic esophageal intramural hematoma includes gastrointestinal disorders such as Mallory–Weiss syndrome, idiopathic esophageal rupture (Boerhaave syndrome), ruptured esophageal varices, esophagitis, and esophageal tumors, as well as non-gastrointestinal conditions such as acute coronary syndrome and aortic disease [1]. Diagnosis is often made by endoscopy and is straightforward when a dark red, smooth-surfaced, longitudinally continuous elevation protruding into the esophageal lumen is present [10, 11]. On CT, IEH typically appears as a relatively homogeneous intramural mass along the esophagus. The presence of a relatively homogeneous, high-density area along the esophagus on CT scan is a strong indicator of this disease [12]. If symptoms are severe, idiopathic esophageal rupture should also be considered, although endoscopy may worsen the patient’s condition [13]. In the present case, the initial absence of mucosal rupture and hematemesis may have delayed endoscopy, thereby contributing to the difficulty in establishing an early diagnosis. The lesion was located in the mid-thoracic esophagus and was characterized by disruption of the muscular layer with a pinhole mucosal perforation. These features correspond to the subtype of idiopathic intramural esophageal hematoma associated with hemothorax that has been described in previous reports. Therefore, despite its atypical presentation, the diagnosis of idiopathic intramural esophageal hematoma in this case is considered appropriate.

Idiopathic intramural hematomas of the esophagus are generally reported to resolve with conservative treatment but are rarely associated with hemothorax [4, 6–9]. Including the present case, six cases of idiopathic esophageal intramural hematoma with hemothorax have been reported to date and are summarized in Table 1. As summarized in Table 1, several reported cases associated with hemothorax were located in the mid- or upper-to-mid thoracic esophagus and showed involvement of the muscular layer or transmural dissection. These findings indicate that such anatomical patterns are not exceptional among severe forms of IEH. Background factors include the following: three patients were taking aspirin; four patients underwent surgery (two with video-assisted thoracoscopic surgery (VATS) and two with open thoracotomy); one patient had an indwelling drain; and one was a fatal case diagnosed at autopsy [8]. Two patients underwent drainage only, one underwent suturing alone, and one underwent esophagectomy. Patients who underwent surgery generally had a favorable course. To our knowledge, excluding the autopsy case, the present case appears to be the only reported patient in whom esophageal perforation was clearly documented, although a previous report described the presence of intestinal bacteria in drainage fluid, suggesting possible microperforation [9]. In the present case, no esophageal perforation was evident at admission; however, a pinhole-like perforation developed during hospitalization. Perforation of the esophagus secondary to thoracic rupture of an idiopathic esophageal intramural hematoma has not been previously reported; this is the first documented case. It is considered that the hematoma within the muscular layer initially ruptured into the thoracic cavity, and the stretched mucosa subsequently developed a pinhole-like perforation under pressure. Idiopathic esophageal intramural hematomas are generally treatable with conservative management, but when accompanied by hemothorax, disruption of the muscular layer should be suspected, and careful observation with video-assisted thoracoscopic surgery (VATS) may be beneficial.

**Table 1:** Idiopathic esophageal intramural hematoma with hemothorax: literature review

Author, year	Age	Sex	Symptom	Aspirin use	Past history	Diagnostic modality	Side of hemothorax	Site of hematoma	Layer with reported disruption	Treatment/intervention	Outcome
Biagi et al [9]	58	Female	Chest pain, shock	Yes	Unknown	Upper GI, Endoscopy	Right	Entire thoracic esophagus	Muscular layer	Thoracotomy, hematoma removal, drainage	Survived
Thumerel et al [7]	63	Female	Chest pain, cough	No	Nephritic colic, osteoporosis	CT, Endoscopy	Right	Upper-to-mid thoracic esophagus	Muscular layer	VATS drainage	Survived
Pomara et al [8]	32	Female	Chest pain, dysphagia, vomiting	No	Neurofibromatosis type 1	Autopsy	Right	Entire thoracic esophagus	Transmural rupture (originating from submucosal hematoma)	None (autopsy case)	Died
Guo et al [6]	51	Male	Chest pain, Vomiting, dyspnea	Yes	Stroke, hypertension	CT, Upper GI	Right	Mid-to-lower thoracic esophagus	Muscular layer	VATS, suturing of muscular tear	Survived
Lin ZX, et al [4]	86	Female	Epigastric pain, vomiting	Yes	Acute lower extremity ischemia	CT, Upper GI, Endoscopy	Left	Upper-to-mid thoracic esophagus	Not specified (intramural hematoma with hemothorax)	Conservative, chest tube drainage	Survived
Present case	56	Male	Chest pain	Yes	Stroke	CT, MRI, Upper GI	Right	Mid-thoracic esophagus	Muscular layer with pinhole mucosal perforation	Open esophagectomy	Survived

We report a rare case of esophageal perforation secondary to idiopathic esophageal intramural hematoma with hemothorax, successfully treated by surgical intervention.

## Abbreviations

IEH: Idiopathic intramural hematoma
CT: Computed tomography
MRI: Magnetic resonance imaging
ICU: Intensive care unit
VATS: Video-assisted thoracoscopic surgery
H&E: Hematoxylin and eosin
UGI: Upper gastrointestinal series
